# Distribution of early-branching Cyanobacteriia and the potential habitats that gave rise to the earliest oxygenic phototrophs

**DOI:** 10.1128/msphere.01013-24

**Published:** 2025-01-30

**Authors:** Christen Grettenberger, David A. Gold, C. Titus Brown

**Affiliations:** 1 Department of Earth and Planetary Sciences, University of California Davis, Davis, California, USA; 2 Department of Environmental Toxicology, University of California Davis, Davis, California, USA; 3 Department of Computer Science and Engineering, Michigan State University, East Lansing, Michigan, USA; E O Lawrence Berkeley National Laboratory, Berkeley, California, USA

**Keywords:** cyanobacteria, evolution, ancestral state reconstruction, bioinformatics, branch water

## Abstract

The evolution of oxygenic photosynthesis in the Cyanobacteria was one of the most transformative events in Earth history, eventually leading to the oxygenation of Earth’s atmosphere. However, it is difficult to understand how the earliest Cyanobacteria functioned or evolved on early Earth in part because we do not understand their ecology, including the environments in which they lived. Here, we use a cutting-edge bioinformatics tool to survey nearly 500,000 metagenomes for relatives of the taxa that likely bookended the evolution of oxygenic photosynthesis to identify the modern environments in which these organisms live. Ancestral state reconstruction suggests that the common ancestors of these organisms lived in terrestrial (soil and/or freshwater) environments. This restricted distribution may have increased the lag between the evolution of oxygenic photosynthesis and the oxygenation of Earth’s atmosphere.

**IMPORTANCE:**

Cyanobacteria generate oxygen as part of their metabolism and are responsible for the rise of oxygen in Earth’s atmosphere over two billion years ago. However, we do not know how long this process may have taken. To help constrain how long this process would have taken, it is necessary to understand where the earliest Cyanobacteria may have lived. Here, we use a cutting-edge bioinformatics tool called branch water to examine the environments where modern Cyanobacteria and their relatives live to constrain those inhabited by the earliest Cyanobacteria. We find that these species likely lived in non-marine environments. This indicates that the rise of oxygen may have taken longer than previously believed.

## OBSERVATION

The evolution of oxygenic photosynthesis was the most transformative evolutionary event in Earth’s history. It eventually led to the oxygenation of Earth’s atmosphere, altering geochemical cycling, metabolic processes, and the evolutionary trajectory of life on Earth. Despite the importance of this event, we know little about the earliest phototrophic Cyanobacteria, which are assumed to be the earliest oxygenic phototrophs, including the environments in which they thrived. However, we must know which environments Cyanobacteria inhabited to properly parameterize models for the timing of the rise of oxygen. Limiting cyanobacterial primary productivity to terrestrial and freshwater environments can increase the timescale for the oxidation of Earth’s atmosphere by at least an order of magnitude (1, 2).

The Cyanobacteria may have emerged in freshwater or non-marine environments based on ancestral state reconstructions using the growth environments of 50 modern Cyanobacteria (3) and the physical characteristics of the early-branching group Gloeobacterales (1). For example, the Gloeobacterales do not survive in marine salinities and have significantly reduced growth in brackish environments under experimental conditions (4). Additionally, modern Gloeobacterales have only been found in terrestrial or freshwater environments (1, 5). However, there are few Gloeobacterales species known; only three are cultivated and an additional seven are known from genomic or metagenomic studies (5–10). Therefore, known Gloeobacterales may not represent the full diversity of the clade, and we must identify as many members of the Gloeobacterales and other early-branching Cyanobacteria as possible and examine their environmental distributions. Recent advances in bioinformatics have allowed large-scale sequence searches of many metagenomes without any additional filtering, reducing the impact of bioinformatics processing such as assembly and binning (11). However, the distribution of the Gloeobacterales and other early-branching Cyanobacteria may not reflect the distribution of the earliest Cyanobacteria. Therefore, it is prudent to also examine the distribution of the Vampirovibrionia (formerly Melainabacteria), the closest non-photosynthetic relative of the Cyanobacteria. Examining the taxa that bookended the evolution of photosynthesis may enable us to infer the environments in which ancient Cyanobacteria lived.

Here, we sought to answer the question: are the taxa that likely bookend the evolution of oxygenic photosynthesis, the early-branching Cyanobacteriia and Vampirovibrionia, restricted to non-marine environments? We chose to use the Gloeobacterales rather than photosynthetic Cyanobacteria (Cyanobacteriia) more broadly because the Gloeobacterales are thought to retain ancestral traits (12), and other early-branching Cyanobacteriia, including the Thermostichales, Pseudanabaenales, and Gloeomargaritales may similarly retain ancestral environmental preferences, while the non-Gloeobacterales Cyanobacteriia diversified rapidly into nearly every illuminated environment on Earth, making it difficult to discern derived versus ancestral environments.

To do so, we retrieved metagenome assembled genomes (MAGs) from the Genomes Taxonomy Database(GTDB) that were classified as Sericytochromatia, Vampirovibronia, Gloeobacterales, Thermostichales, Pseudanabaenales, and Gloeomargaritales. We retained genomes that were >70% complete. The final data set included 218 genomes from Sericytochromatia (*n* = 14), Vampirovibrionia (*n* = 151) Gloeobacterales (*n* = 13), Thermostichales (*n* = 9), Pseudanabaenales (*n* = 30), and the Gloeomargaritales (*n* = 6) to search 498,942 publicly available metagenomic data sets in the National Center for Biotechnology Information (NCBI) using branch water (https://branchwater.jgi.doe.gov, https://github.com/sourmash-bio/sra_search/commit 3253ffe), a k-mer-based approach for content-based search of metagenomes (11, 13). Metagenomes that had >20% containment (~94% average nucleotide similarity to the target genome) (11) of one or more genomes or MAGs were retained. To create the character matrix, metagenomes were classified as host-associated, wastewater, freshwater, brackish, marine, hydrothermal (including hot springs), soil, plant-associated, or “other/unknown” based on the “scientific name” classification in NCBI, which, for environmental samples, records the environment from which the sample was taken. If the environment was unclear, the metagenome was found in NCBI and classified based on the additional metadata available in NCBI or the associated publication(s). Metagenomes from synthetic data and those for which an environment could not be determined were removed. Genomes for which there were no metagenome hits were removed (*n* = 5). We constructed a multiple sequence alignment of the remaining genomes by collecting 71 single-copy marker genes using the “Bacteria_71” gene set in anvi’o (14, 15) and aligning them in anvi’o using muscle (16).

A maximum likelihood phylogenetic tree was generated using IQTree v.2.2.2.6 (17). Ancestral state reconstruction was performed on the tree with corHMM v.2.8 (18) and Phytools v.2.0.13 (19). Each environment was tested separately as a presence/absence binary and analyzed separately. For each character, we tested four different models: (i) an equal-rate model, (ii) an all-rates-different model, and (iii and iv) each model with two additional hidden rates. We used analysis of variance to determine the best-fitting model and then performed stochastic character mapping using the preferred model and 1,000 simulations. The list of species, metagenome hits, code, and results from the tree-building and ancestral state reconstruction analyses are available on GitHub (https://github.com/DavidGoldLab/2024_Cyano_ASR).

By querying our genomes against metagenomic databases, we were able to identify the suite of environments these organisms inhabit. The early-branching Cyanobacteriia genomes were primarily found in freshwater (51.2%) and hydrothermal (40.3%) environments. The high proportion of hydrothermal environments was driven by the Thermostichales, which were found there 98% of the time (Table 1). The early-branching Cyanobacteriia were rarely found in marine environments (0.2%). The earliest branching group, the Gloeobacterales, was found predominantly in freshwater environments, including from the Arctic (6), Antarctic (20), and Canada, and one metagenome was from a hot spring environment (21). None of these environments were marine. The Vampirovibrionia were found primarily in host-associated metagenomes (94.5% of instances) or those from wastewater (4.7% of instances) and were found less commonly in freshwater (0.5% of instances), brackish (<0.01% of instances), marine environments (0.1% of instances), and hydrothermal environments (<0.1% of instances). The Sericytochromatia were found predominately in freshwater (23%), soil and rock (33%), and plant-associated environments (29.9%). They were rarely found in marine environments (1.4%) and never in brackish environments. Twenty-nine taxa account for all instances in marine and brackish environments, whereas 92 taxa are found in freshwater environments.

**TABLE 1:** Number of genomes used, number of total hits, and proportion of hits for each environmental type for each order-level clade^*a*^

Taxonomy	Order	No. of genomes	No. of total hits	Freshwater (%)	Brackish (%)	Marine (%)	Hydrothermal (%)	Soil and rock (%)	Plant associated (%)	Host associated (%)	Wastewater (%)	Cultivated (%)	Unknown (%)	Other (%)
Cyanobacteriia	Gloeobacterales	13	85	45.9	2.4	–	2.4	48.2	**–**	–	–	–	1.2	–
	Gloeomargaritales	6	17	<0.1	17.6	–	82.4	–	–	–	–	–	–	–
	Pseudanabaenales	24	753	90.2	5.7	–	1.3	0.1	0.4	0.1	1.2	–	0.9	–
	Thermostichales	6	546	–	1.1	0.5	98.4	–	–	–	–	–	–	–
	**Total**	**49**	**1,401**	**51.2**	**3.8**	**0.2**	**40.3**	**3.0**	**0.2**	**<0.1**	**0.6**	**–**	**0.6**	**–**
Sericytochromatia	JACMPN01	1	47	–	–	4.3	4.3	10.6	80.9	–	–	–	–	–
	S15B-MN24	7	143	23.1	–	0.7	1.4	46.2	17.5	–	–	0.7	3.5	7.0
	UBA7694	5	34	24.8	–	0.7	1.4	44.8	17.2	–	–	0.7	3.4	6.9
	**Total**	**13**	**224**	**23.0**	**–**	**1.4**	**3.2**	**33.0**	**29.9**	**–**	**–**	**0.4**	**3.2**	**4.5**
Vampirovibronia	2–02-FULL-35–15	2	117	83.8	–	–	–	16.2	–	–	–	–	–	–
	Caenarcaniphilales	7	74	87.8	–	8.1	–	<0.1	–	2.7	1.4	–	–	–
	Gastranaerophilales	104	173,140	0.1	<0.1	–	–	<0.1	–	98.6	1.3	–	<0.1	–
	LMEP-6097	3	163	33.7	8.6	57.7	–	–	–	0.0	–	–	–	–
	Obscuribacterales	28	7,250	8.3	<0.1	1.3	0.3	1.3	0.1	0.9	87.3	–	0.4	–
	Vampirovibrionales	7	27	44.4	–	–	–	3.7	11.1	–	29.6	11.1	–	–
	**Total**	**151**	**180,771**	**0.50**	**<0.1**	**0.10**	**<0.1**	**<0.1**	**<0.1**	**94.50**	**4.70**	**<0.1**	**<0.1**	**–**

^*a*^ Dashes indicate that there were no hits for the clade. Food and air environments are not shown because they never accounted for more than 0.5% of the hits for any group. Bold text indicates the total for each class level group.

**Fig 1:**
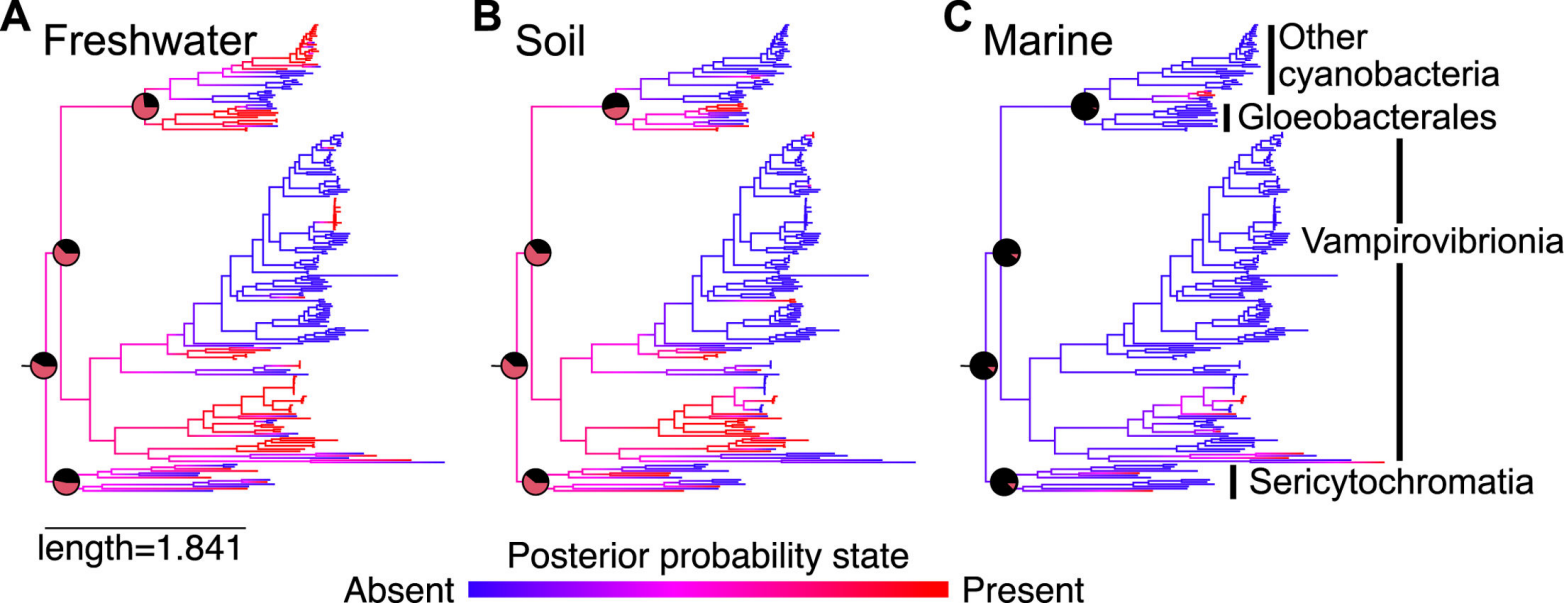
Summary of ancestral state reconstruction for select environments. The trees are visualized as density maps, with several important nodes visualized through pie charts. All three analyses represent the “all-rates-different” model without hidden rates, which was the preferred model of evolution. (**A**) Freshwater, (**B**) soil, and (**C**) marine.

Based on these data, none of the taxa examined commonly inhabit marine environments. Ancestral state reconstruction suggests that both the common ancestor of Vampirovibrionia, Sericytochromatia, and Cyanobacteriia inhabited freshwater (posterior probability = 0.578) and/or soil environments (posterior probability = 0.616) (Fig. 1). All other environments had less than 50% posterior probability at the ancestral node, and in most cases—including marine settings—the probability was close to zero (posterior probability [marine] = 0.116). Therefore, we predict that the first photosynthetic Cyanobacteria inhabited non-marine environments and that Cyanobacteriia began inhabiting marine and brackish environments only after the divergence of Gloeomargaritales. When this assumption is used in geochemical models, the oxygenation of Earth’s atmosphere took ~1 million years (1) rather than less than 100,000 years (2). This work is based on modern Cyanobacteria and their available genomes. These species have undergone billions of years of evolution since they diverged from their common ancestor, and there is likely undiscovered diversity within these clades on modern Earth, both of which introduce potential error in our work. However, our conclusion is robust across multiple early-branching cyanobacterial clades and is therefore a good approximation of the evolutionary history of this clade.

This work shows the strength of using tools like branch water that enable rapid, large-scale content-based sequence search of unfiltered data. However, these tools are hindered by data sets that are not annotated in detail in public data sets. For example, we hand-curated thousands of entries where the “Scientific Name” is listed as “metagenome” rather than the species or environment. These poor annotations will prevent larger-scale comparisons from being done in the future.

## Data Availability

All the data used here are from publicly available repositories including the National Center for Biotechnology Information Sequence Read Archive. and the Genome Taxonomy Database. The code and results from these analyses are available on GitHub (github.com/DavidGoldLab/2024_Cyano_ASR).

## References

[1] Raven JA, Sánchez-Baracaldo P. 2021. Gloeobacter and the implications of a freshwater origin of Cyanobacteria. Phycologia 60:402–418. doi:10.1080/00318884.2021.1881729

[2] Ward LM, Kirschvink JL, Fischer WW. 2016. Timescales of oxygenation following the evolution of oxygenic photosynthesis. Orig Life Evol Biosph 46:51–65. doi:10.1007/s11084-015-9460-3 26286084

[3] Blank CE, Sánchez-Baracaldo P. 2010. Timing of morphological and ecological innovations in the Cyanobacteria--a key to understanding the rise in atmospheric oxygen. Geobiology 8:1–23. doi:10.1111/j.1472-4669.2009.00220.x 19863595

[4] Herrmann AJ, Gehringer MM. 2019. An investigation into the effects of increasing salinity on photosynthesis in freshwater unicellular Cyanobacteria during the late archaean. Geobiology 17:343–359. doi:10.1111/gbi.12339 30874335

[5] Grettenberger CL. 2021. Novel Gloeobacterales spp. from diverse environments across the globe. mSphere 6:e0006121. doi:10.1128/mSphere.00061-21 34287010 PMC8386580

[6] Pessi IS, Popin RV, Durieu B, Lara Y, Tytgat B, Savaglia V, Roncero-Ramos B, Hultman J, Verleyen E, Vyverman W, Wilmotte A. 2023. Novel diversity of polar Cyanobacteria revealed by genome-resolved metagenomics. Microb Genom 9:9. doi:10.1099/mgen.0.001056 PMC10438808 37417735

[7] Rahmatpour N, Hauser DA, Nelson JM, Chen PY, Villarreal A JC, Ho M-Y, Li F-W. 2021. A novel thylakoid-less isolate fills a billion-year gap in the evolution of Cyanobacteria. Curr Biol 31:2857–2867. doi:10.1016/j.cub.2021.04.042 33989529

[8] Rippka R, Waterbury J, Cohen-Bazire G. 1974. A cyanobacterium which lacks thylakoids. Arch Microbiol 100:419–436. doi:10.1007/BF00446333

[9] Saw JH, Cardona T, Montejano G. 2021. Complete genome sequencing of a novel gloeobacter species from a waterfall cave in Mexico. Genome Biol Evol 13:evab264. doi:10.1093/gbe/evab264 34850891 PMC8691054

[10] Saw JHW, Schatz M, Brown MV, Kunkel DD, Foster JS, Shick H, Christensen S, Hou S, Wan X, Donachie SP. 2013. Cultivation and complete genome sequencing of gloeobacter Kilaueensis sp. nov., from a lava cave in Kīlauea Caldera, Hawai’i. PLoS One 8:e76376. doi:10.1371/journal.pone.0076376 24194836 PMC3806779

[11] Lumian J, Sumner D, Grettenberger C, Jungblut AD, Irber L, Pierce-Ward NT, Brown CT. 2022. Biogeographic distribution of five Antarctic Cyanobacteria using large-scale k-mer searching with sourmash branchwater. Biorxiv. doi:10.1101/2022.10.27.514113 PMC10909832 38440141

[12] Mimuro M, Tomo T, Tsuchiya T. 2008. Two unique cyanobacteria lead to a traceable approach of the first appearance of oxygenic photosynthesis. Photosynth Res 97:167–176. doi:10.1007/s11120-008-9311-4 18568415

[13] Pierce NT, Irber L, Reiter T, Brooks P, Brown CT. 2019. Large-scale sequence comparisons with sourmash. F1000Res 8:1006. doi:10.12688/f1000research.19675.1 31508216 PMC6720031

[14] Eren AM, Esen ÖC, Quince C, Vineis JH, Morrison HG, Sogin ML, Delmont TO. 2015. Anvi’o: an advanced analysis and visualization platform for ’omics data. PeerJ 3:e1319. doi:10.7717/peerj.1319 26500826 PMC4614810

[15] Campbell JH, O’Donoghue P, Campbell AG, Schwientek P, Sczyrba A, Woyke T, Söll D, Podar M. 2013. UGA is an additional glycine codon in uncultured SR1 bacteria from the human microbiota. Proc Natl Acad Sci USA 110:5540–5545. doi:10.1073/pnas.1303090110 23509275 PMC3619370

[16] Edgar RC. 2004. MUSCLE: multiple sequence alignment with high accuracy and high throughput. Nucleic Acids Res 32:1792–1797. doi:10.1093/nar/gkh340 15034147 PMC390337

[17] Nguyen L-T, Schmidt HA, von Haeseler A, Minh BQ. 2015. IQ-TREE: a fast and effective stochastic algorithm for estimating maximum-likelihood phylogenies. Mol Biol Evol 32:268–274. doi:10.1093/molbev/msu300 25371430 PMC4271533

[18] Beaulieu JM, O’Meara BC, Donoghue MJ. 2013. Identifying hidden rate changes in the evolution of a binary morphological character: the evolution of plant habit in campanulid angiosperms. Syst Biol 62:725–737. doi:10.1093/sysbio/syt034 23676760

[19] Revell LJ. 2012. Phytools: an R package for phylogenetic comparative biology (and other things). Methods Ecol Evol 3:217–223. doi:10.1111/j.2041-210X.2011.00169.x

[20] Grettenberger CL, Sumner DY, Wall K, Brown CT, Eisen JA, Mackey TJ, Hawes I, Jospin G, Jungblut AD. 2020. A phylogenetically novel cyanobacterium most closely related to gloeobacter. ISME J 14:2142–2152. doi:10.1038/s41396-020-0668-5 32424249 PMC7368068

[21] Reichart NJ, Bowers RM, Woyke T, Hatzenpichler R. 2021. High potential for biomass-degrading enzymes revealed by hot spring metagenomics. Front Microbiol 12:668238. doi:10.3389/fmicb.2021.668238 33968004 PMC8098120

